# Problematic Internet, Smartphone, and SMS Use among Adults: Shared and Unique Predictors

**DOI:** 10.34172/jrhs.2022.97

**Published:** 2022-12-29

**Authors:** Argyroula Kalaitzaki, Stéphanie Laconi, George Tsouvelas

**Affiliations:** ^1^Social Work Department, Health Sciences Faculty, Hellenic Mediterranean University; Laboratory of Interdisciplinary Approaches to the Enhancement of Quality of Life (Quality of Life Lab); Affiliated Researcher of the University Research Centre ‘Institute of AgriFood and Life Sciences’, Greece; ^2^Laboratoire CERPPS (Centre d’Études et de Recherches en Psychopathologie et Psychologie de la Santé) - EA 7411 - Université Toulouse 2 Jean Jaurès, UFR de Psychologie, Bureau M153, 5 Allée Antonio Machado, 31058 TOULOUSE Cedex, 9. France; ^3^Department of Nursing, University of West Attica; Affiliated researcher of the Laboratory of Interdisciplinary Approaches for the Enhancement of Quality of Life, Hellenic Mediterranean University, Greece

**Keywords:** Addiction, Internet, Overuse, Personality disorders, Smartphone, SMS text

## Abstract

**Background:** Although a surge of interest has recently emerged in investigating the simultaneous problematic use of various technology-based tools, the findings are still inconclusive. The present web-based survey aimed at examining whether (a) personality traits, coping strategies, and sociodemographics are associated with problematic internet, smartphone, and SMS use among Greek users and (b) personality traits mediate the relationship between maladaptive coping strategies and problematic use of the three media.

**Study Design:** A cross-sectional study.

**Methods:** A convenience and snowball sample of 1016 participants (84.4% female, mean age of 30.3 years) completed the Problematic Internet Use Questionnaire-9 (PIUQ-9), the Mobile Phone Problem Use Scale (MPPUS), the Self-Perception of Text Message Dependency Scale (STDS), the Personality Diagnostic Questionnaire 4+(PDQ-4+), and the Brief Coping Orientation to Problems Experienced Inventory (Brief COPE).

**Results:** Shared predictors between problematic use of the three technology-based tools were younger age and low educational level, the coping strategy of substance use, and the narcissistic, avoidant, and dependent personality disorders. Predictors of problematic internet and smartphone use were coping strategies of emotional support, self-distraction, and behavioral disengagement. According to structural equation models (SEM) models, cluster C personality disorders fully mediate the relationship between maladaptive coping strategies and problematic use of technology-based tools.

**Conclusion:** Addressing factors that predispose (i.e., personality traits), precipitate, or maintain problematic use (i.e., coping strategies) can lead to effective and cost-saving preventive (i.e., screening of vulnerable groups) and therapeutic efforts (i.e., teaching adaptive coping strategies).

## Background

 Α surge of interest has emerged over the past two decades in examining the problematic overuse of technology-based tools due to their detrimental repercussions on individual mental health and well-being.^1-6^

 Problematic internet use (PIU) and problematic smartphone use (PSU) have received much attention from researchers. PIU and PSU can be both defined as one’s inability to control the internet^7^ or smartphone^8^ with negative repercussions in daily life. As the internet and smartphone are interrelated (i.e., the ubiquity of internet access has increased smartphone ownership and vice versa), similarly, problematic use of both seems to simultaneously occur. Taufik et al^9^ found that high school students with PIU have a 60% chance of having PSU. Problematic text messaging (PTM) is related to PSU. PTM may be defined as “impaired control over texting, intense emotional reactions (anxiety, frustration, feelings of rejection) arising from texting, high importance of messaging in social life, and negative consequences secondary to this behavior.”^10^

 Commonalities or similar underlying mechanisms should be found between these problematic technology-related behaviors to ensure a better understanding of these concepts. However, most studies only focus on one of these media and very few have simultaneously examined the problematic use of multiple tools, typically, two of them (either PIU and PSU or PSU and PTM). Personality disorders and maladaptive coping strategies have received the most attention among the factors that have been related to problematic technology use, though a consensus fails to exist in the literature.

 Amendola et al^1^ found an intercorrelation between problematic technology use and overall personality dysfunction in adolescents and Phillips and Shipps^11^ found a relationship with the personality traits of introversion and neuroticism. In their systematic review, Moor and Anderson^12^ investigated that several problematic technology-related behaviors were associated with psychopathy, and Machiavellianism and everyday sadism were consistently related to these behaviors. In a cross-cultural study conducted in 14 countries, Laconi et al^13^ found PIU to be predicted by narcissistic, histrionic, antisocial (Cluster B), dependent and avoidant (Cluster C) personality traits and negatively by schizoid and paranoid traits (Cluster A). PSU has been associated with neuroticism and impulsivity,^14^ though the effect sizes were small to moderate.^15^ Kalaitzaki et al^16^ have shown dependent (Cluster C), histrionic, and narcissistic personality disorders (Cluster B) to be consistent predictors of PSU in the overall and most of the 14 samples.

 Studies have consistently shown that maladaptive coping strategies were associated with problematictechnologyuse.Vally et al^17^ have found that PIU was positively predicted by maladaptive coping strategies and negatively by adaptive coping in young adult users in the United Arab Emirates. In large cross-cultural studies, maladaptive coping was a positive predictor of both PIU^13^ and PSU.^16^ The specific maladaptive coping strategies implicated in the problematic use of each technology tool need to be examined.

 The present study aimed to examinewhether (a) personality traits, coping strategies, and sociodemographics are associated with PIU, PSU, and PTM among Greek users and (b) personality traits mediate the relationship between maladaptive coping strategies and the latent variable of problematicuse of the three technology-based media. The conceptual model of this study incorporates predisposing (i.e., personality traits) and precipitating or maintaining factors (i.e., coping strategies) of problematic use of the three media. It was assumed that those who differ in certain personality traits and use different coping strategies are likely to have problematic use of different media.

## Methods

 The present study was a cross-sectional web-based survey conducted in Greece.

###  Participants

 A total of 1037 Greek participants were initially recruited from the general population. A final sample of 1016 participants remained after excluding unusual cases with anomaly index, 10% missing data, no mobile phone, and participants under 18. Overall, 21 (2%) participants were excluded mainly due to missing values which randomly occurred as no specific pattern was detected regarding missing values.

###  Measures 

 Participants responded to sociodemographic questions (e.g., gender, age, professional status, educational level, and marital status), internet, phone, and SMS use-related questions (e.g., hours spent per day), and validated questionnaires. All scales were in Greek.^13^ Cronbach alphas can be seen in Table 1.

**Table 1:** Descriptive statistics and reliabilities

**Variables**	**Min**	**Max**	**Mean**	**SD**	**Items**	**Cronbach's α**
Problematic smartphone use	27	216	102.7	33.6	27	0.89
Problematic text messaging	15	72	33.3	11.3	13	0.90
Problematic internet use	9	43	19.1	7.0	9	0.88
COPE Active	2	8	5.9	1.2	2	0.63
COPE Planning	2	8	6.2	1.3	2	0.67
COPE Instrumental Support	2	8	5.7	1.5	2	0.83
COPE Emotional Support	2	8	5.7	1.5	2	0.76
COPE Venting	2	8	5.4	1.4	2	0.55
COPE Positive Reframing	2	8	6.0	1.4	2	0.76
COPE Acceptance	2	8	5.6	1.3	2	0.38
COPE Humour	2	8	4.4	1.5	2	0.58
COPE Religion	2	8	3.8	1.8	2	0.75
COPE Self-Distraction	2	8	5.5	1.4	2	0.50
COPE Denial	2	8	3.7	1.4	2	0.61
COPE Self-blame	2	8	5.2	1.5	2	0.69
COPE Substance Use	2	8	2.5	1.1	2	0.92
COPE Behavioral disengagement	2	8	3.1	1.2	2	0.73
PDQ Paranoid	0	7	3.4	1.7	7	0.54
PDQ Histrionic	0	8	2.7	1.6	8	0.46
PDQ Antisocial	0	6	0.8	1.0	8	0.47
PDQ Obsessive-Compulsive	0	8	3.7	1.6	8	0.38
PDQ Narcissistic	0	9	2.9	1.7	9	0.54
PDQ Avoidant	0	7	2.5	1.8	7	0.67
PDQ Borderline	0	9	2.7	2.0	9	0.67
PDQ Dependent	0	7	1.5	1.6	8	0.65
PDQ Schizotypal	0	9	3.1	1.9	9	0.58
PDQ Schizoid	0	6	1.7	1.3	7	0.42
PDQ Negativistic	0	7	2.3	1.7	7	0.60
PDQ Depressive	0	7	3.3	1.8	7	0.62

Abbreviations: PDQ, Personality Diagnostic Questionnaire; COPE, Coping Orientation to Problems Experienced.

*Problematic use measures: *PSU was measured with a 27- item Mobile Phone Problem Use Scale (MPPUS).^13,18^ PIU was assessed with the Problematic Internet Use Questionnaire-9 (PIUQ-9).^13,19^ PTM was assessed with a 15-item Self-Perception of Text Dependency Scale(STDS).^13,20^ Higher scores on each scale indicate more problematic use.

*Personality and Psychopathology measures: *Coping strategies were evaluated with the Brief Coping Orientation to Problems Experienced Inventory (COPE),^13,21^ comprising 28 items allocated in 14 subscales. Pathological personality traits were assessed with the Personality Diagnostic Questionnaire 4 + (PDQ-4 + ),^13,22^ comprising 99 items, allocated in three clusters.

###  Procedure 

 A google-forms questionnaire was distributed using convenience and snowball sampling procedures. Participants were recruited through email contacts and social networking sites, and they were, in turn, asked to recruit their contacts similarly. The first page of the questionnaire contained information about the study and informed consent. The study conforms with the 1964 Helsinki Declaration and its later amendments and received approval from the ethics committee of the Hellenic Mediterranean University (number UVT8170/16.04.2018).

###  Data analysis

 Anomaly detection models were used to identify outliers or unusual cases. Cases with anomaly index value greater than 2 were considered anomaly candidates.^23^ Internal consistency was examined with Cronbach’s alpha. Three hierarchical multiple regression analyses (using the stepwise method) were performed to identify predictors of PIU, PSU, and PTM by sociodemographic factors (age, gender, marital status, and educational level were introduced in the first step), coping strategies, and personality traits (were introduced in the second step). All analyses with *P* < 0.05 were considered significant and were performed with IBM SPSS v23. Following the regression findings, two structural equation models were conducted with AMOS v20 using the maximum-likelihood estimation method to separately test the mediating effect of B and C personality clusters in the relationship between maladaptive coping strategies and the latent variable of problematic use of technology-based tools (comprising PIU, PSU, and PTM). Parametric bootstrapping of standard errors across 2000 samples was used for the estimation of indirect effects. Model fit indices were assessed^24,25^: the SRMR less than 0.08, the TLI, the comparative fit index above 0.90, and finally, the RMSEA less than 0.06.^25^

## Results

###  Descriptive statistics 

 The final sample was mostly women (84.4%), middle-aged (30.3 years old), employed (49.7%), well-educated (75.3%), and in a relationship (54%) (Table 2). Participants presented high mean scores on PSU (10.27), PTM (33.3), and PIU (19.1). They used more frequently the coping strategies of ‘planning’ (6.2) and ‘positive reframing’ (6.0) and less frequently the ‘behavioral disengagement’ (3.1) and ‘substance use’ (2.5). They had the highest PDQ4 mean scores on ‘obsessive-compulsive’ **(**3.7) and the lowest mean scores on ‘antisocial’ personality (0.80) (Table 2).

**Table 2:** Sociodemographic characteristics of the participants

**Categorical variables**	**Number**	**Percent**
Gender		
Male	159	15.6
Female	857	84.4
Professional status		
University student	398	39.2
Employed	505	49.7
Unemployed	113	11.1
Educational level		
University	43	4.2
Master’s degree	762	75.3
Doctoral degree	207	20.5
Marital status		
Single	467	46.0
In a relationship	549	54.0
**Continuous variables**	**Mean**	**SD**
Age (y)	30.3	10.5

###  Predictors of PIU, PSU, and PTM

 Regression analyses (see Table 3) showed that PIU was negatively predicted by age, educational level, marital situation (singles presented higher scores), and paranoid trait, and positively by the personality traits narcissistic, avoidant, dependent, depressive, and the coping strategies of self-distraction, substance use, and behavioral disengagement. PSU was negatively predicted by age, educational level, and schizoid personality traits, and positively by the personality traits of narcissistic, avoidant, borderline, dependent, schizoid, and negativistic, and the coping strategies of emotional support, self‒distraction, substance use, and behavioral disengagement. PTM was negatively predicted by age, educational level, and marital status (singles presented higher scores), and positively by gender (women presented higher scores) the personality traits histrionic, narcissistic, avoidant, and dependent and by the coping strategies of emotional support, humor, denial, substance use.

**Table 3:** Hierarchical multiple regression analyses for predicting problematic smartphone use, problematic text messaging, and problematic internet use by sociodemographic factors, coping strategies, and personality traits

	**PSU**					**PTM**					**PIU**				
**Variables**	**Step**	**B**	**SE**	**b**	***P *** **value**	**Step**	**B**	**SE**	**b**	***P *** **value**	**Step**	**B**	**SE**	**b**	***P *** **value**
Age (y)	1	‒0.56	0.09	‒0.17	0.001	1	‒0.37	0.03	‒0.33	0.001	1	‒0.07	0.02	‒0.11	0.001
Gender (1 = male, 0 = female)						2	3.34	0.82	0.11	0.001					
Marital status (0 = single, 1 = in a relationship)						3	‒1.37	0.61	‒0.06	0.025	2	‒0.85	0.38	‒0.06	0.026
Educational level	2	‒3.03	2.03	‒0.04	0.136	4	‒1.43	0.65	‒0.06	0.028	3	‒0.92	0.41	‒0.06	0.027
COPE Emotional Support	6	2.14	0.63	0.10	0.001	8	0.59	0.20	0.08	0.003					
COPE Humour						10	0.39	0.19	0.05	0.045					
COPE Self‒Distraction	4	2.20	0.68	0.09	0.001						7	0.51	0.13	0.10	0.001
COPE Denial						7	0.74	0.23	0.09	0.001					
COPE Substance Use	9	2.34	0.83	0.08	0.005	12	0.59	0.27	0.06	0.027	8	0.54	0.17	0.09	0.001
COPE Behavioral disengagement	7	2.08	0.83	0.08	0.012						10	0.47	0.17	0.08	0.005
PDQ Paranoid											11	‒0.30	0.13	‒0.07	0.026
PDQ Schizoid	11	‒2.12	0.78	‒0.08	0.007										
PDQ Histrionic						6	0.60	0.22	0.08	0.006					
PDQ Narcissistic	8	1.92	0.61	0.10	0.002	11	0.51	0.20	0.08	0.011	9	0.43	0.13	0.10	0.001
PDQ Avoidant	10	1.83	0.67	0.10	0.006	5	0.84	0.20	0.13	0.001	5	0.55	0.13	0.14	0.001
PDQ Dependent	3	3.84	0.73	0.18	0.001	9	0.59	0.23	0.08	0.013	4	0.90	0.15	0.21	0.001
PDQ Obsessive-Compulsive											6	0.54	0.14	0.12	0.001
PDQ Negativistic	5	1.70	0.69	0.09	.013										
PDQ Depressive															
R^2^	0.27					0.34					0.29				

Abbreviations: PDQ, Personality Diagnostic Questionnaire; COPE, Coping Orientation to Problems Experienced; PSU, problematic smartphone use; PTM, problematic text messaging; PIU, problematic internet use. Note: Step = each step shows the order in which each predictor was entered into the model based on the stepwise method. Empty cells indicate that the predictor did not contribute significantly to the dependent variables, and it was not included in the model. B = Unstandardized Beta values; b = Standardized beta values. The dotted lines define the first step in hierarchical multiple regression at which the effect of demographic factors was examined. The indicators in the table are those of the final regression.

###  Mediation of B and C personality clusters between maladaptive coping strategies and problematic use of the three technology-based tools

**Figure 1:**
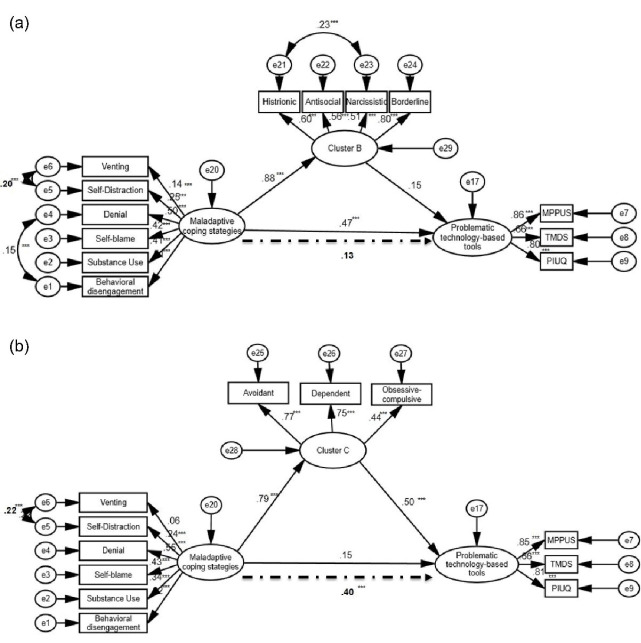
Mediating effects of (a) cluster B personality traits and (b) cluster C personality traits in the relationship between maladaptive coping strategies and problematic use of technology-based tools. The standardized path coefficients are presented. Dotted lines refer to indirect effects.

 Based on the results of the regression analyses, two structural equation models examined the mediating effect of B and C personality clusters separately in the relationship between maladaptive coping strategies and the latent variable of problematic use of technology-based tools (comprising of PIU, PSU, and PTM). The mediation model for Cluster B demonstrated an acceptable fit (CMIN = 271.09, DF = 59, *P* < 0.001; CFI = 0.94; IFI = 0.94; TLI = 0.92; RMSEA = 0.06 [LO = 0.05, HI = 0.06]; SRMR = 0.04). However, the path between Cluster B and problematictechnologyuse was not significant (0.15, *P* = 0.596) and, therefore, a mediation model could not be supported (see Figure 1a). The mediation model for Cluster C demonstrated acceptable fit (CMIN = 219.11, DF = 50, *P* < 0.001; CFI = 0.95; IFI = 0.93; TLI = 0.93; RMSEA = 0.06 [LO = 0.05, HI = 0.06]; SRMR = 0.04). When entering the mediator (cluster C), the direct effect of maladaptive coping strategies on problematic technology use was no longer significant but it was the indirect effect through the mediator. The coefficients for the direct effect were 0.15, *P* = 0.123 and for the indirect effect 0.40, *P* < 0.001, suggesting a full mediation (see Figure 1b).

## Discussion

 To the best of our knowledge, this is the only study that examines shared predictors between problematic use of three technology-based tools (i.e., internet, smartphone, and SMS). Although the co-occurrence of various technologies has been examined in terms of the prevalence rates, no study has explored shared predictors between them. It is well-known, though, that adults commonly use more than one technological instrument simultaneously with varying degrees of involvement and/or potential problematic use of each.^1^ The findings of the present study provided evidence for shared and unique predictors of problematic technology uses.

 In line with the previous findings,^2^ younger age was associated with more problematic use of the three technology tools. Young people seem to resort to the virtual world to enable socialization and self-expression rather than engaging in face-to-face interchanges.^26^ Low education level was another shared predictor. Also, people with low educational levels might have limited other resources to relieve stress than over-engagement with technology.

 Concerning maladaptive personality functioning, the present study revealed that narcissistic (Cluster B), avoidant, and dependent personality traits (Cluster C) were shared predictors of the problematic use of the three technology-based tools (i.e., PIU, PSU, and PTM). The results of the mediation analysis also corroborated the significance of Cluster C personality disorders in indirectly exacerbating the problematic use of the three media. Literature has separately associated these personality traits with one at a time of these media. PIU (e.g., Facebook use) has been associated with narcissistic or avoidant personality,^17,26-28^ and PSU has been associated with narcissistic, avoidant, or dependent personality.^16,29-32^ The pathologic users of the three media seem to combine characteristics of the three personality traits. They plausibly have avoidant traits of resorting to virtual life because they try to avoid distressing experiences, discomfort, loneliness, or other difficulties in real life,^7,31,33^ dependent traits of over-engagement as a means to maintain relationships because of their fear of separation/being left alone^28^ and their need to be reassured, and narcissistic traits of self-presentation through these media (e.g., posting and uploading photos). Both avoidant and dependent personalities share a fear of being criticized or rejected in social situations.^28^

 As expected, individuals with less schizoid traits made more PSU, those with less paranoid traits made more PIU, and ones with more histrionic traits made more PTM. The less socially detached people with fewer deficiencies in emotional expression (schizoid traits) engage in PSU and the less suspicious and distrustful (paranoid traits) engage in PIU. Besides, attention seeking (histrionic traits) has been associated with vaguebooking (i.e., intentionally vague posts aiming at eliciting emotional support or gaining attention from other users).^34,35^

 Substance use, as a coping strategy,was also a shared predictor of problematic use of the three technology tools. Behavioral addiction literature has shown that people who overuse technology (e.g., such as those with internet addiction), have similar brain scans to those who are addicted to substances (e.g., alcohol, cocaine, and cannabis).^36^ Besides, Mellouli et al^37^ have shown a potentially bidirectional association between poor control of internet use and lifetime tobacco and illicit drug use.

 Many other dysfunctional or maladaptive coping strategies were distinctively associated with PIU, PSU, and PTM; emotional support, self-distraction, and behavioral disengagement predicted PIU and PSU, whereas denial predicted PTM. Substance use,self-distraction, denial, and behavioral disengagement seem to represent escape and avoidance strategies, and these findings corroborate the assumption that individuals may resort to overuse of these media to deny reality or distract themselves from unwanted reality and/or quit efforts to cope with it. These strategies may have a positive function in eliminating, suppressing, or discharging stress, hurtful thoughts, and emotions (thus buffering from long-term detrimental mental health outcomes). The finding that emotional support was associated with PSU and PTM is in line with this assertion.

 The limitations of this study should be acknowledged. The cross-sectional nature of the study permits reference only to associations. The convenience sample of mostly women and privileged participants (e.g., well-educated) disqualifies the representativeness of the sample and the external validity of the study. Low internal consistency indices of the Brief COPE and PDQ4 may have resulted in measurement error. The self-reported data collected online may have resulted in under or over-reporting of problematic use and selection bias, respectively.

 Despite the limitations, the implications of this study are worth noting. The value of this study is in recognizing and proposing shared and unique factors associated with the problematic use of three technology-based tools and indicating the target group (i.e., young with low education and substance use as coping) to which policy efforts should be guided. Therefore, it seems that several intervention and prevention guidelines could be common among problematic users of the three media, whereas many others could be differentially effective.

 Given that reducing the time spent fails to be effective,^38^ national campaigns and early interventions in Greece should target young people and poorly educated, and also modifiable factors associated with problematic technology use, such as increasing awareness about dysfunctional coping strategies and teaching adaptive ones. Addressing shared factors among problematic users of the three media could be national cost and resource-saving. Health professionals and clinicians should be aware that cluster C personality disorders amplify the relationship between maladaptive coping strategies and problematic use in treating adults.

## Conclusion

 Overall, the present study provides useful insights into the role of personality dysfunction and maladaptive coping strategies used by adults with problematic use of the internet, smartphone, and SMS, highlighting the importance of addressing shared and unique variables, both predisposing and precipitating or maintaining ones, in developing and implementing treatment and preventive efforts.

**Highlights**

- Maladaptive coping, cluster B and C personality traits predicted problematic use
- Younger age and low educational level predicted problematic use of the three media
- Cluster C personality traits amplified the problematic use of the three media
- Investigation of shared factors among problematic users of the three media is cost-saving
- Protection of at-risk populations with adaptive coping strategies is important

## Acknowledgments

 The authors would like to express their sincere gratitude to all the participants in this study and appreciate their time.

## Conflict of interest

 All authors declare that there is no conflict of interest regarding the publication of the present study.

## Funding

 The authors received no financial support for the research, authorship, and/or publication of this article.

## References

[1] Amendola S, Spensieri V, Biuso GS, Cerutti R (2020). The relationship between maladaptive personality functioning and problematic technology use in adolescence: a cluster analysis approach. Scand J Psychol.

[2] Busch PA, McCarthy S (2021). Antecedents and consequences of problematic smartphone use: a systematic literature review of an emerging research area. Comput Human Behav.

[3] Aznar-Díaz I, Kopecký K, Romero-Rodríguez JM, Cáceres-Reche MP, Trujillo-Torres JM (2020). Pathologies associated with problematic internet use. A systematic review and meta-analysis in WoS and Scopus. Investig Bibl.

[4] Sohn SY, Rees P, Wildridge B, Kalk NJ, Carter B (2019). Prevalence of problematic smartphone usage and associated mental health outcomes amongst children and young people: a systematic review, meta-analysis and GRADE of the evidence. BMC Psychiatry.

[5] Thomée S (2018). Thomée SMobile phone use and mental healthA review of the research that takes a psychological perspective on exposure. Int J Environ Res Public Health.

[6] Wacks Y, Weinstein AM (2021). Excessive smartphone use is associated with health problems in adolescents and young adults. Front Psychiatry.

[7] Spada MM (2014). An overview of problematic internet use. Addict Behav.

[8] Billieux J, Maurage P, Lopez-Fernandez O, Kuss DJ, Griffiths MD (2015). Can disordered mobile phone use be considered a behavioral addiction? An update on current evidence and a comprehensive model for future research. Curr Addict Rep.

[9] Taufik JR, Tiatri S, Allida VB (2021). Problematic Smartphone Use and Problematic Internet Use: The Two Faces of Technological Addiction. Adv Heal Sci Res.

[10] Spritzer DT, Andrade ALM, Xavier AZ, da Silva GT, Kim HS, Kaliszewska-Czeremska K, et al. The Self-perception of Text message Dependence Scale (STDS): a Brazilian-Portuguese validation and expansion of its psychometric properties. Curr Psychol. 2022:1-12. 10.1007/s12144-022-02957-8. PMC8914152 35291222

[11] Phillips B, Shipps B (2022). Problematic technology use: the impact of personality and continued use. J South Assoc Inf Syst.

[12] Moor L, Anderson JR (2019). A systematic literature review of the relationship between dark personality traits and antisocial online behaviours. Pers Individ Diff.

[13] Laconi S, Kalaitzaki A, Spritzer DT, Hauck S, Gnisci A, Sergi I, et al. A Cross-cultural exploration of problematic Internet use, pathological personality traits, defense mechanisms, coping strategies, and self-esteem in 14 countries. Annales Médico-psychologiques, revue psychiatrique. 2022. 10.1016/j.amp.2022.09.008.

[14] De-Sola Gutiérrez J, Rodríguez de Fonseca F, Rubio G (2016). Cell-phone addiction: a review. Front Psychiatry.

[15] de Francisco Carvalho L, Sette CP, Ferrari BL (2018). Problematic smartphone use relationship with pathological personality traits: systematic review and meta-analysis. Cyberpsychology: Journal of Psychosocial Research on Cyberspace.

[16] Kalaitzaki A, Laconi S, Spritzer DT, Hauck S, Gnisci A, Sergi I, et al. The prevalence and predictors of problematic mobile phone use: a 14-country empirical survey. Int J Ment Health Addict. 2022. 10.1007/s11469-022-00901-2.

[17] Vally Z, Laconi S, Kaliszewska-Czeremska K (2020). Problematic internet use, psychopathology, defense mechanisms, and coping strategies: a cross-sectional study from the United Arab Emirates. Psychiatr Q.

[18] Bianchi A, Phillips JG (2005). Psychological predictors of problem mobile phone use. Cyberpsychol Behav.

[19] Koronczai B, Urbán R, Kökönyei G, Paksi B, Papp K, Kun B (2011). Confirmation of the three-factor model of problematic internet use on off-line adolescent and adult samples. Cyberpsychol Behav Soc Netw.

[20] Igarashi T, Motoyoshi T, Takai J, Yoshida T. The text messaging addiction scale: factor structure, reliability, and validity. In: Sixth Biennial Conference of the Asian Association of Social Psychology; 2005; Wellington, New Zealand.

[21] Carver CS (1997). You want to measure coping but your protocol’s too long: consider the brief COPE. Int J Behav Med.

[22] Hyler SE. Personality Diagnostic Questionnaire-4 (PDQ-4). New York: New York State Psychiatric Institute; 1994.

[23] International Business Machines Corporation (IBM). Anomaly Detection. IBM; 2021. Available from: https://www.ibm.com/docs/en/igfa/10.0.0?topic=system-anomaly-detection. Accessed March 8, 2021.

[24] Hooper D, Coughlan J, Mullen MR (2008). Structural equation modelling: guidelines for determining model fit. Electron J Bus Res Methods.

[25] Hu LT, Bentler PM (1999). Cutoff criteria for fit indexes in covariance structure analysis: conventional criteria versus new alternatives. Struct Equ Modeling.

[26] Laconi S, Vigouroux M, Lafuente C, Chabrol H (2017). Problematic internet use, psychopathology, personality, defense and coping. Comput Human Behav.

[27] Casale S, Fioravanti G (2018). Why narcissists are at risk for developing Facebook addiction: the need to be admired and the need to belong. Addict Behav.

[28] Verseillié É, Laconi S, Chabrol H (2020). Pathological traits associated to Facebook and Twitter among French users. Int J Environ Res Public Health.

[29] Alavi SS, Ghanizadeh M, Farahani M, Jannatifard F, Esmaili Alamuti S, Mohammadi MR (2020). Addictive use of smartphones and mental disorders in university students. Iran J Psychiatry.

[30] Direktör C, Nuri C (2019). Personality beliefs as a predictor of smartphone addiction. Arc Clin Psychiatr.

[31] Gorday JY, Bardeen JR (2022). Problematic smartphone use influences the relationship between experiential avoidance and anxiety. Cyberpsychol Behav Soc Netw.

[32] Pearson C, Hussain Z (2015). Smartphone use, addiction, narcissism, and personality: a mixed methods investigation. Int J Cyber Behav Psychol Learn.

[33] Şenormancı Ö, Konkan R, Güçlü O, Şenormancı G (2014). Evaluation of coping strategies of male patients, being treated in internet addiction outpatient clinic in Turkey. J Mood Disord.

[34] Berryman C, McHugh B, Wisniewski P, Ferguson C, Negy C. User characteristics of vaguebookers versus general social media users. In: Meiselwitz G, ed. Social Computing and Social Media Design, Human Behavior and Analytics. Cham: Springer; 2019. p. 169-81. 10.1007/978-3-030-21902-4_13.

[35] Buehler EM (2017). “You shouldn’t use Facebook for that”: navigating norm violations while seeking emotional support on Facebook. Soc Media Soc.

[36] Goldstein RZ, Volkow ND (2011). Dysfunction of the prefrontal cortex in addiction: neuroimaging findings and clinical implications. Nat Rev Neurosci.

[37] Mellouli M, Zammit N, Limam M, Elghardallou M, Mtiraoui A, Ajmi T (2018). Prevalence and predictors of internet addiction among college students in Sousse, Tunisia. J Res Health Sci.

[38] Šporčić B, Glavak-Tkalić R (2018). The relationship between online gaming motivation, self-concept clarity and tendency toward problematic gaming. Cyberpsychology: Journal of Psychosocial Research on Cyberspace.

